# Drug resistance related to aberrant glycosylation in colorectal cancer

**DOI:** 10.18632/oncotarget.22377

**Published:** 2017-11-03

**Authors:** Ninon Very, Tony Lefebvre, Ikram El Yazidi-Belkoura

**Affiliations:** ^1^ Unité de Glycobiologie Structurale et Fonctionnelle, UGSF-UMR 8576 CNRS, Université de Lille, Lille 59000, France

**Keywords:** drug therapy resistance mechanisms, cancer chemotherapy, cancer-associated glycosylations, glycosyltransferases, colorectal cancer

## Abstract

Colorectal cancer (CRC) is the fourth leading cause of cancer-related deaths in the world. Drug resistance of tumour cells remains the main challenge toward curative treatments efficiency. Several epidemiologic studies link emergence and recurrence of this cancer to metabolic disorders. Glycosylation that modifies more than 80% of human proteins is one of the most widepread nutrient-sensitive post-translational modifications. Aberrant glycosylation participates in the development and progression of cancer. Thus, some of these glycan changes like carbohydrate antigen CA 19-9 (sialyl Lewis a, sLea) or those found on carcinoembryonic antigen (CEA) are already used as clinical biomarkers to detect and monitor CRC. The current review highlights emerging evidences accumulated mainly during the last decade that establish the role played by altered glycosylations in CRC drug resistance mechanisms that induce resistance to apoptosis and activation of signaling pathways, alter drug absorption and metabolism, and led to stemness acquisition. Knowledge in this field of investigation could aid to the development of better therapeutic approaches with new predictive biomarkers and targets tied in with adapted diet.

## INTRODUCTION

According to the World Health Organization (WHO) [1], colorectal cancer (CRC) is the third most common diagnosed cancer and the fourth cause of cancer-related death in the world (1,3 million new cases and 694 000 deaths respectively in 2012). When diagnosed at an early stage, surgical resection is curative for most of the cases but for advanced stages a survival advantage is gained with the systemic administration of cytotoxic chemotherapy (5-year recurrence rates after primary surgery are nearly 10% and 36% for early (stages 1 and 2A of the tumor/node/metastasis (TNM) classification of malignant tumors) and late (stages 2B and 3) stage diseases respectively [2]). One established chemotherapy regimen for the treatment of advanced CRC (from stages 2B to 4) consists of the combination of 5-fluorouracil (5-FU)/leucovorin (LV, folinic acid) with oxaliplatin or irinotecan (camptothecin-11, CPT-11) [3]. Furthermore, targeted therapy based on the use of the monoclonal antibodies cetuximab and panitumumab that specifically bind the epidermal growth factor receptor (EGFR) and bevacizumab that neutralizes the vascular endothelial growth factor (VEGF) confers a benefit when administered in conjunction with chemotherapy [3]. However, the response rate is only about 57% [4] and the 5-year survival rate remains around 12,5% for patients with metastatic-stage disease [5]. The failure of treatments is primarily due to the development of drug resistance. In this context, much of current research is focused on the understanding of cancer resistance mechanisms and the identification of new predictive biomarkers and targets for drug therapy. We notice a renewed interest in the study of glycosylation since it was established that, compared to adjacent non-cancerous cells, CRC cells display glycosylation alterations which correlate with cancer progression and resistance to drug treatments. This review addresses the role of glycans in drug resistance mechanisms in CRC therapy. Particularly, the involvement of glycosyltransferases and related glycosylations in biological mechanisms controlling CRC development, progression and drug therapy resistance will be discussed.

## THE MULTIPLE FACES OF GLYCOSYLATION IN MAMMALIAN CELLS

**Figure 1:**
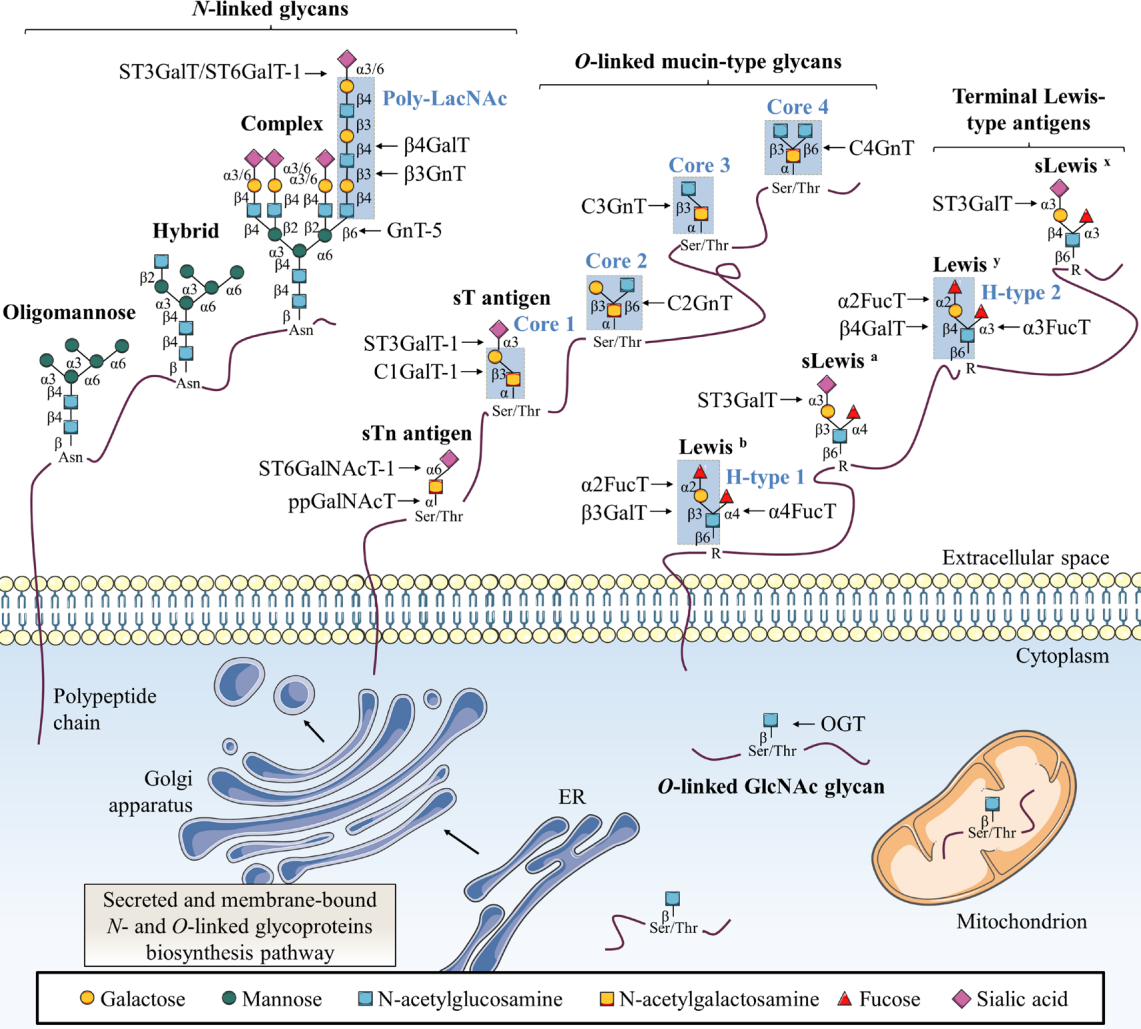
Classical glycosylation types found on mammalian proteins This figure depicts common *N*-linked and *O*-linked glycoprotein structures as well as terminal Lewis antigens structures. The key glycosyltransferases responsible for the addition of specific glycans are also indicated. Secreted and membrane-bound glycoproteins exhibit *N*-glycans with β-GlcNAc linked to Asn as oligomannose, complex or hybrid forms. *O*-glycans are linked through α-GalNAc to Ser/Thr with various core structures and extensions. Terminal structures of *N*-linked and *O*-linked glycans are often sialylated and fucosylated to generate Lewis-type antigen glycosylation. Single β-*O*-GlcNAc is found on many cytosolic, nuclear and mitochondrial proteins.

Glycosylation is a group of post-translational modifications (PTM) in which carbohydrates are enzymatically linked to proteins, carbohydrates, lipids or any other kind of molecule [6]. Glycosylation represents one of the most abundant PTM since nearly 1–2% of human genome encode 236 different glycosyltransferases (CAZy database) and more than 80% of human proteins are glycosylated [7]. This modification can affect the folding, stability, subcellular localization, partners interaction and biological activity of a glycoprotein. Major protein glycosylations are classified according to the atom that links the glycan to the aglycone:nitrogen of the amide group of asparagine (Asn, N) for *N*-linked glycosylation (*N*-glycosylation) and oxygen of the hydroxyl group of serine (Ser, S) or threonine (Thr, T) for *O*-linked glycosylation (*O*-glycosylation) (Figure 1).

### *N*-glycosylation

In the *N*-linked glycosylation biosynthetic pathway, the preassembled precursor oligosaccharide GlcNAc2Man9Glc3 is co-translationally transferred from the dolichol pyrophosphate donor to an asparagine residue located in a NXS/T consensus sequence (where X is any amino-acid except proline (Pro, P)) of the nascent polypeptide in the lumen of the rough *endoplasmic reticulum* (ER) where *N*-glycosylation is necessary for protein folding and quality control [8]. This glycosylation is further processed and extended in the Golgi apparatus into oligomannose, hybrid or complex structures (Figure 1). N-acetylglucosaminyltransferases (GnT) are enzymes responsible for *N*-glycan branching. Beta1,6-branched *N*-glycans can be elongated with a poly-N-acetyllactosamine (poly-LacNAc) chain by the sequential addition of Galβ1,4-GlcNAcβ1,3 moieties by β1,4-galactosyltransferases (β4GalT) and β1,3-N-acetylglucosaminyltransferases (β3GnT). Alpha2,3-sialyltransferases (ST3GalT) and β-galactoside α2,6-sialyltransferase 1 (ST6GalT-1) mediate the α2,3- and α2,6-sialylation of complex *N*-linked glycoproteins [9].

### Mucin-type *O*-glycosylation

Two of the most representative *O*-linked glycosylations are *O*-linked α-N-acetylgalactosamine (*O*-GalNAc) commonly found in mucins and *O*-linked β-N-acetylglucosamine (*O*-GlcNAc). Mucin-type *O*-glycosylation is initiated by post-translationally transfer of N-acetylgalactosamine (GalNAc) through an α-linkage in the Golgi [9]. Then, *O*-glycans are elongated with other carbohydrates to build various cores (Figure 1). Terminal structures of *N*-linked and *O*-linked mucin-type glycans are often sialylated and fucosylated and can generate Lewis (Le) blood group antigen structures. Mucin-type *O*-glycosylation is initiated by a large family of Golgi polypeptide N-acetylgalactosaminyltransferases (ppGalNAcT) [10] which transfer a GalNAc residue from uridine-5’-diphosphate-N-acetylglucosamine (UDP-GalNAc) to serine or threonine residues to generate the Thomsen-nouvelle (Tn) antigen. Then, Tn antigen can be extended by sequential enzymatic reactions to form four core structures (Figure 1). Core 1 β1,3-galactosyltransferase 1 (C1GalT-1) enzyme catalyzes the synthesis of core 1 (also named Thomsen-Friedenreich antigen, T antigen) by transferring a galactose (Gal) residue in a β1,3 linkage to the Tn antigen, and core 2 β1,6-N-acetylglucosaminyltransferases (C2GnT) generate the core 2 by branching a GlcNAc residue in a β1,6 linkage to core 1. Both Tn and T antigens can be further sialylated into sialyl-Tn antigen (sTn) and sialyl-T antigen (sT) respectively by α-GalNAc α2,6-sialyltransferase 1 (ST6GalNAcT-1) and ST3GalT-1 causing premature termination of chain elongation. As an alternative to core 1, core 3 β1,3-N-acetylglucosaminyltransferase (C3GnT) adds a GlcNAc residue in a β1,3 linkage to the Tn antigen to form the core 3. Finally, like for core 1, core 3 can be extended into core 4 by the core 4 N-acetylglucosaminyltransferase (C4GnT) which transfers another GlcNAc residue in a β1,6 linkage [9]. As *N*-glycosylation, mucin-type *O*-glycosylation takes place in the ER and/or Golgi apparatus biosynthetic-secretory pathway and targets secreted or membrane-bound proteins. Based on their cellular localization, mucins (MUC) are classified as secretory or membrane-bound glycoproteins. Membrane bound mucins (MUC1, MUC3-4, MUC12-17 and MUC20) are anchored to the apical membrane of epithelial cells by a transmembrane domain and are involved in signal transduction. In contrast, secretory mucins (MUC2, MUC5AC, MUC5B, MUC6-8 and MUC19) lack the transmembrane domain and are secreted into the extracellular space to compose a viscoelastic mucus gel, a protective and lubricative molecular barrier [11].

### Lewis-type antigen glycosylation

Depending on the core disaccharide linkage on *N*- or *O*-linked glycoproteins, terminal Lewis blood group antigens are classified as either type 1 (Galβ1,3-GlcNAc) or type 2 (Galβ1,4-GlcNAc) structures [9, 12]. Alpha1,2-fucosyltransferases (α2FucT) 1 and 2 (FucT-1 and FucT-2) transfer a fucose (Fuc) in an α1,2 linkage to type 1 and type 2 structures to produce respectively H1- and H2-type determinants (Figure 1). H1- and H2-type structures can be further converted into Lewis b (Leb) and Lewis y (Ley) di-fucosylated antigens respectively by fucosylation in an α1,4 or an α1,3 linkage by α1,3/4-fucosyltransferases (α3/4FucT) FucT-3-7 and FucT-9 [9, 12]. Lewis a (Lea) and Lewis x (Lex) mono-fucosylated antigens are positional isomers of respectively Leb and Ley antigens but are not fucosylated in α1,2. Moreover, α1,4- and α1,3-fucosylation of Lea and Lex can be forewent by the addition of α2,3-sialic acid by ST3GalT-3-4 and ST3GalT-6 to form sialyl Lea (sLea) and sialyl Lex (sLex) [9, 12].

### *O*-GlcNAcylation

Contrary to *N*- and mucin-type *O*-glycosylations found in secreted and membrane-anchored glycoproteins, *O*-GlcNAcylation occurs exclusively on cytoplasmic, nuclear and mitochondrial proteins [13]. *O*-GlcNAcylation is also different from all other classical glycosylations since it consists in the reversible addition of a single GlcNAc residue (Figure 1). It is a dynamic PTM that implies addition and removal of GlcNAc residues by a unique couple of antagonist enzymes, *O*-GlcNAc transferase (OGT) and *O*-GlcNAcase (OGA) respectively. OGT transfers a single GlcNAc from uridine-5’-diphosphate-N-acetylglucosamine (UDP-GlcNAc) via a β1-linkage to hydroxyl group of serine or threonine residues of intracellular proteins involved in a plethora of biological processes such as cell signaling, transcription or cell cycle [13–16].

## FROM THE METABOLISM SHIFT TO THE ALTERATIONS OF GLYCOSYLATIONS IN CANCER CELLS

### Warburg effect and hexosamine biosynthetic pathway

**Figure 2:**
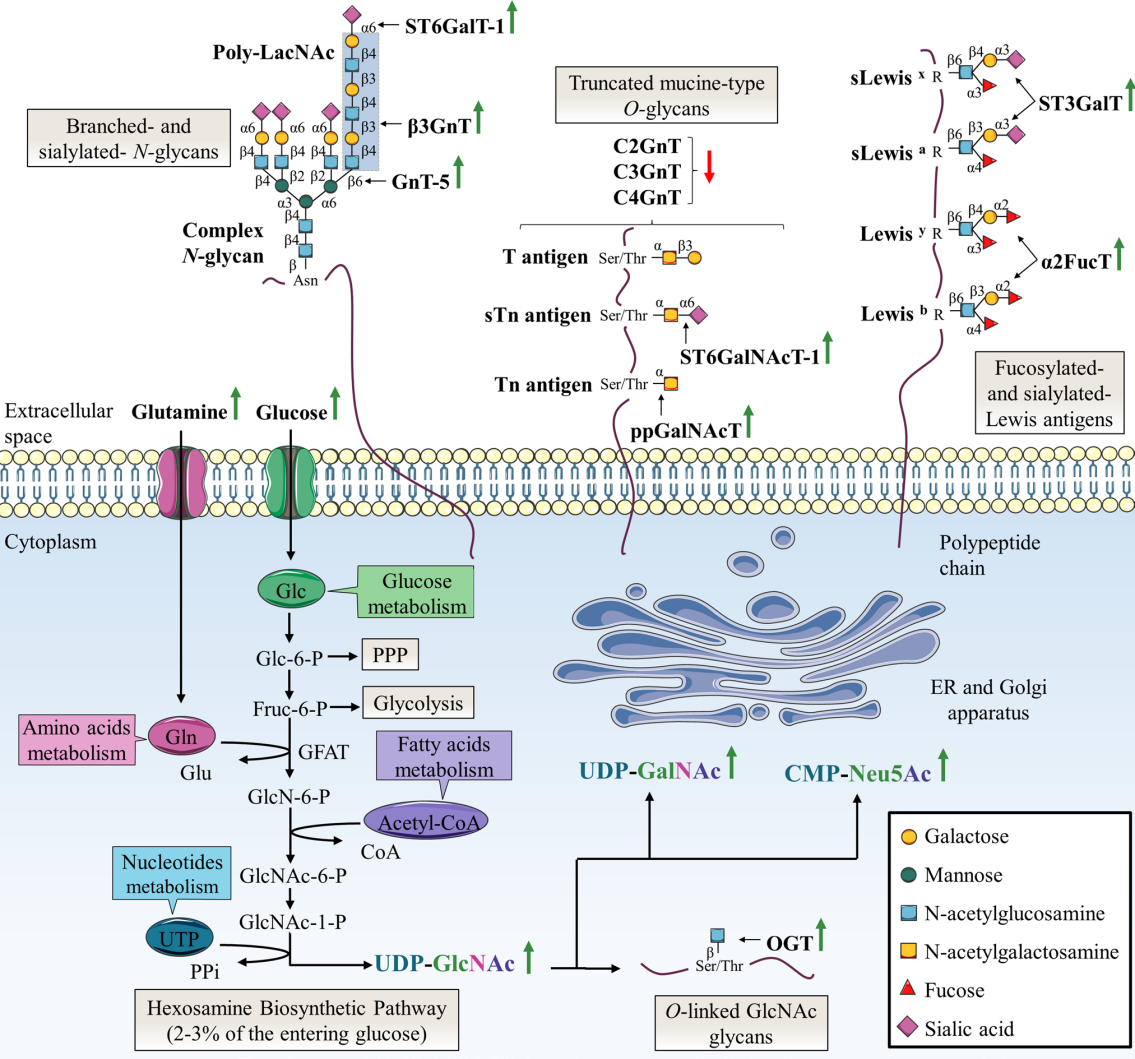
Specific alterations of glycosylation in colorectal cancer The HBP pathway produces the nucleotide sugar UDP-GlcNAc in a nutrient-dependent manner. UDP-GlcNAc is critical for most kinds of glycosylation including *N*-glycosylation, *O*-GalNAc-based glycosylation and *O*-GlcNAcylation. In this sense, glycan structures are modified according to the metabolic status of the cell. The abnormal glycosylation which occurs in cancer cells can be attributed to abundance and availability of nucleotide sugars, acceptor substrates or cofactors in the same way as altered expression or activity, or mislocalization of glycosyltransferases. The most frequent abnormal glycosylations observed in CRC are increased branched- and sialylated-*N*-glycans, truncated mucin-type *O*-glycans, increased fucosylation and sialylation of Lewis antigens and increased *O*-GlcNAcylation. Green and red arrows indicate respectively increased and decreased availability of nutrients and nucleotide sugars, or expression or activity of glycosyltransferases in CRC. Acetyl-coA: acetyl-coenzyme A; Fru-6-P: fructose-6-phosphate; Glc-6-P: glucose-6-phosphate; GlcN-6-P: glucosamine-6-phosphate; GlcNAc-6-P: N-acetylglucosamine-6-phosphate; GlcNAc-1-P: N-acetylglucosamine-1-phosphate; Glu: glutamic acid; UTP: uridine triphosphate; PPi: inorganic pyrophosphate.

One of the key hallmarks of cancer cells is the Warburg effect, an adaptive metabolic shift from oxidative phosphorylation to aerobic glycolysis [17, 18]. This adaptive metabolic is thought to provide an evolutionary advantage to cancer cells by providing both increase bioenergetics and biosynthesis. Warburg effect is characterized by an increase of glucose (Glc) and glutamine (Gln) consumption. Glucose and glutamine are the most abundant extracellular nutrients which support the growth and proliferation of cancer cells by contributing to energy production (glycolysis via glucose and Krebs cycle via glutaminolysis) and *de novo* biosynthesis of macromolecules (lipids, nucleic acids and proteins) [19, 20]. It is possible to take advantage of the increased cancer cells demand to detect primary and metastatic tumor sites by monitoring the incorporation of the glucose radioanalogue 2-deoxy-2-(18F)fluoroglucose ([18F]FDG]) with positron emission tomography (PET) scan [21]. Elevated glucose uptake takes an active part in the increase of the pentose phosphate pathway (PPP) to produce reduced nicotinamide adenine dinucleotide phosphate (NADPH2) needed for fatty acid synthesis and pentoses incorporated in nucleic acids, and the hexosamine biosynthetic pathway (HBP) that generates UDP-GlcNAc (Figure 2). Approximately 2-3% of the glucose entering the cell are directed to the HBP [22]. Glutamine takes also an active part in HBP as the first and rate limiting step of this pathway is catalyzed by glutamine:fructose-6-phosphate amidotransferase (GFAT) which converts fructose-6-phosphate (Fru-6-P) to glucosamine-6-phosphate (GlcN-6-P) using glutamine as the amine group donor [23]. Through a subset of enzymatic reactions, GlcN-6-P is then converted to the nucleotide sugar UDP-GlcNAc, the end-product of HBP. UDP-GlcNAc is considered as a nutritional state sensor of the cell because it integrates glucose, amino acids, fatty acids and nucleotides metabolisms. UDP-GlcNAc can undergo epimerization to generate UDP-GalNAc used in the ER and Golgi apparatus and cytidine-5’-monophospho-N-acetylneuraminic acid (CMP-Neu5Ac), the donor of Neu5Ac, used in the Golgi apparatus for terminal glycosylation of cell membrane and secreted glycoproteins (Figure 2).

### Loss of glycosylation control in cancer cells

Another well-established cancer phenotypic marker is altered glycosylation that underlies tumor growth and malignancy. Specific aberrant glycosylations are associated to CRC. These glycosylation abnormalities are caused by increased abundance and availability of nucleotide sugars [24–26] and altered expression or activity of the corresponding glycosyltransferases [27]. Specifically, overexpression of OGT is correlated with overall increased *O*-GlcNAcylation of intracellular proteins [28]. In the other hand, overexpression of GnT-5 [29] and β3GnT [30] is responsible for an increase of branched-*N*-glycans with respectively GlcNAc residue and poly-LacNAc chain. Truncated mucin-type *O*-glycans such as T and Tn antigens result from the overexpression of the first-step biosynthesis enzymes ppGalNAcT combined to down-expression of *O*-glycan extension enzymes C2GnT, C3GnT and C4GnT [31–33]. Increased activity of α2FucT participate also in the synthesis of Leb and Ley oncofetal antigens [34–37]. These antigens are expressed in colon during fetal life, gradually decrease in adult life to be mostly restricted to the proximal colon and are re-expressed in distal colon and rectal carcinomas [34, 37]. Finally, the most-widely aberrant glycosylation associated with CRC is an increase of global sialylation. Overexpression of sialyltransferases such as ST6GalT-1, ST3GalT and ST6GalNAcT-1 is involved in aberrant glycan structures including α2,6-sialylated *N*-glycans, sTn, sLea (also known as carbohydrate antigen 19-9, CA 19-9) and sLex (Figure 2). SLea and carcinoembryonic antigen (CEA) are widely used as serum glycoprotein biomarkers to monitor and detect recurrence of CRC even if they show low specificity and sensitivity in initial diagnosis [38]. Recently, targeted next-generation sequencing defined the mutational landscapes of glycosylation-associated genes in CRC. Notably, authors identified somatic mutations in glycosyltransferase genes encoding β3GnT-2, β4GalT-2 and ST6GalNAcT-2 involved in the synthesis of poly-LacNAc chains on *N*- and *O*-glycans for the two former, and in the sialylation-mediated termination chain elongation of *O*-glycan core 1 and 3 structures for the latter. Functional studies showed that these mutations are responsible for impairment of localization, enzymatic activity and PTM pattern [39].

In addition to have a major role in the CRC development and progression, some of these aberrant glycosylations have been correlated to drug resistance by interfering with metabolism, absorption, anti-proliferative and anti-apoptotic effects of the drugs. This will be discussed in detail in the next sections by type of glycosylation.

## GLYCOSYLATION ALTERATIONS IN COLORECTAL CANCER CELLS AND RESISTANCE TO DRUG THERAPY

### *N*-glycosylation alterations in colorectal cancer: a cause of drug resistance

#### Disturbing *N*-glycosylation profile impacts the response to chemotherapy

**Figure 3:**
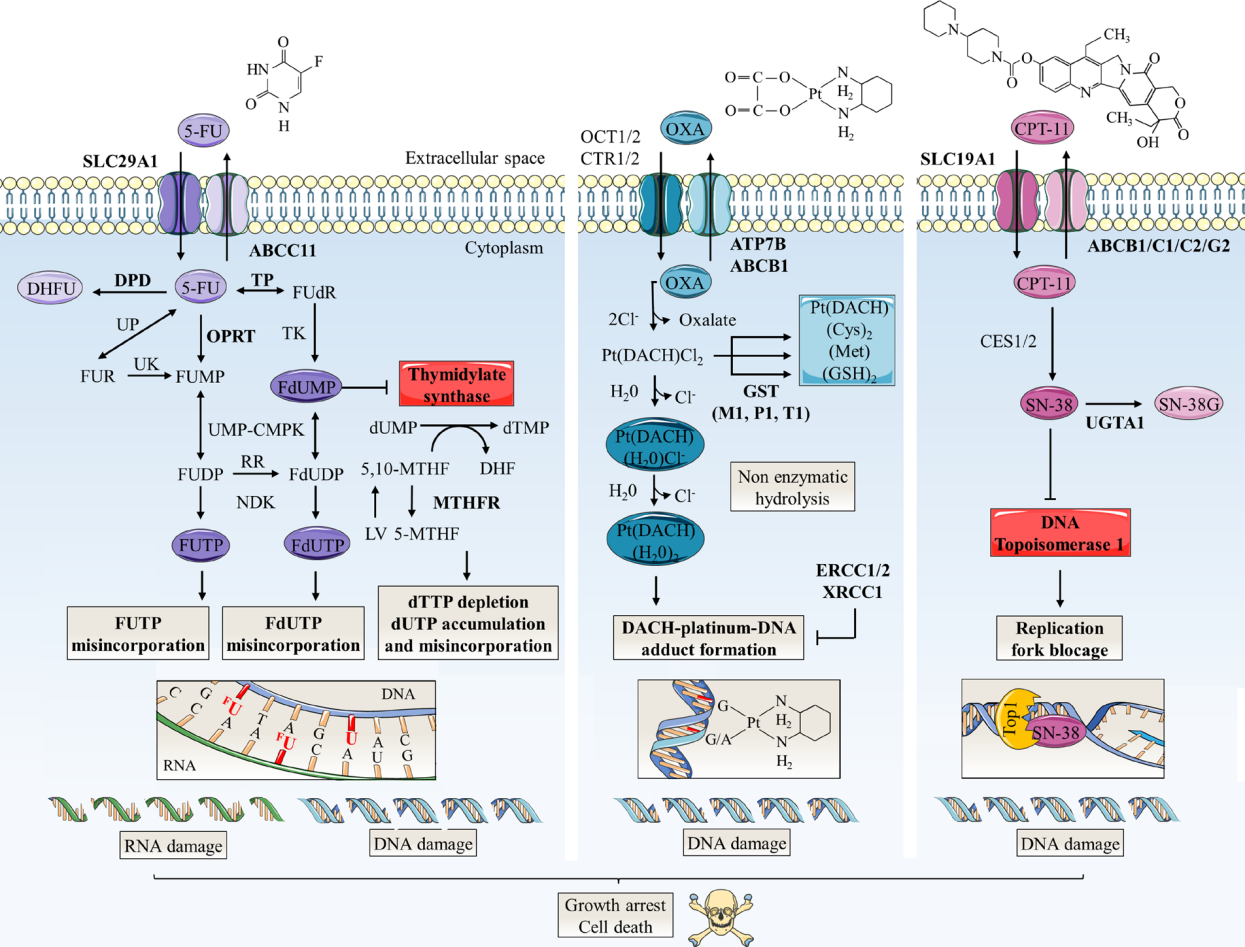
Schematic representation of mechanisms of action and resistance biomarkers in main colorectal cancer chemotherapies 5-FU, oxaliplatin (OXA) and CPT-11 are the principal anti-colorectal cancer drugs. Arrow-headed lines indicate metabolite chemical conversion whereas bar-headed lines represent inhibition of chemical process (OXA pathway) or enzymes (5-FU and CPT-11 pathways). The anabolic pathways are dark-colored and the catabolic pathways are light-colored. Some of therapy resistance biomarkers are indicated in bold. 5-FU penetrates in tumor cell by nucleoside solute carrier (SLC) transporters such as SLC29A1. Then, 5-FU is converted to three main active metabolites: FdUMP, fluorodeoxyuridine triphosphate (FdUTP) and fluorouridine triphosphate (FUTP) which acts as antimetabolites and pyrimidine analogues. FdUMP decreases the biosynthesis of pyrimidine nucleotides by inhibiting TS, the enzyme that catalyzes the rate limiting step in DNA synthesis by catalysing the reductive methylation of deoxyuridine monophosphate (dUMP) to deoxythymidine monophosphate (dTMP) using 5,10-methylenetetrahydrofolate (5,10-MTHF) as the methyl donor. By this way, FdUMP inhibits DNA synthesis and repair leading to DNA strand breakage and cell death notably by induction of apoptosis. 5-FU is also incorporated into DNA (via FdUTP) or RNA (via FUTP), leading to other cytotoxic actions including DNA fragmentation and decrease in protein synthesis. LV expands the intracellular pool of 5,10-MTHF and increases the toxicity of 5-FU. DPD mediates the conversion of 5-FU to non-active dihydrofluorouracil (DHFU). 5-FU catabolism also includes efflux of metabolites by ABC transporters such as ABCC11. TS and OPRT over-expressions are major molecular mechanism of 5-FU resistance. OXA, a third generation of alkylating platinum agent, is transported mainly by organic cation (OCT1/2) and copper (CTR1/2) transporters. On the contrary, P-type ATPase (ATP7A/B) transporters and ABC transporters promote its efflux. Inside the cell, displacement of the labile oxalate and non-enzymatic hydrolysis promotes the conversion of OXA in active metabolites such as monoaquo-1,2-diaminocyclohexane (DACH) platinum and diaquo-DACH platinum. These products alkylate DNA leading to G/G or G/A intra-strand crosslinks which, if not repaired, will block both DNA replication and transcription leading to apoptosis. Hydrophobicity and bulkiness of the DACH ring prevents the MMR proteins from binding to OXA. Excision repair cross-complementation 1 and 2 (ERCC1/2) and X-ray repair cross complementing 1 (XRCC1) are involved in repair of DNA adducts. Cellular detoxification processes include metabolites targeting for excretion by conjugation of aquated compounds to cysteine (Cys), methionine (Met) or GSH. In particular, conjugation of GSH is catalysed by GST (-mu GSTM1, -pi GSTP1 and -theta GSTT1). CPT-11 is transported by SLC transporters such as SLC19A1. Inside the cell, CPT-11 is converted into the active SN-38 by carboxylesterases 1 and 2 (CES1/2). SN-38 binds and stabilizes its target DNA Top1 responsible for supercoiled DNA relaxation during replication and transcription. SN-38 inhibits the relegation step and the collision of the SN-38/Top1 complex with the moving DNA replication fork leads to irreversible arrest of the replication fork and double DNA stranded breaks. This damage causes cell-cycle arrest and apoptosis. CPT-11 resistance mechanisms include SN-38 glucuronidation in inactive SN-38 glucuronide (SN-38G) by uridine diphosphate glucuronosyltransferase 1 polypeptide A1 (UGT1A1) and overexpression of ABC transmembrane transporters (ABCB1, ABCC1, ABCC2 and ABCG2) responsible for efflux of SN-38. 5-MTHF: 5-methyltetrahydrofolate; Cl-: chlorine; DHF: dihydrofolate; dTTP: deoxythymidine triphosphate; dUTP: deoxyuridine triphosphate; FdUDP: fluorodeoxyuridine diphosphate; FU: fluorouracil; FUDP: fluorouridine diphosphate; FUMP: fluorouridine monophosphate; FUR: fluorouridine; MTHFR: methylene tetrahydrofolate reductase; NDK: nucleoside-diphosphate kinase; Pt(DACH)(Cl2): dichloro-DACH platinum; Pt(DACH)(H20)2: diaquo-DACH platinum; Pt(DACH)(H20)Cl-: monoaquo-DACH platinum; RR: ribonucleotide reductase; UK: uridine kinase; UMP-CMPK: uridine monophosphate/cytidine monophosphate kinase; UP: uridine phosphorylase.

**Figure 4:**
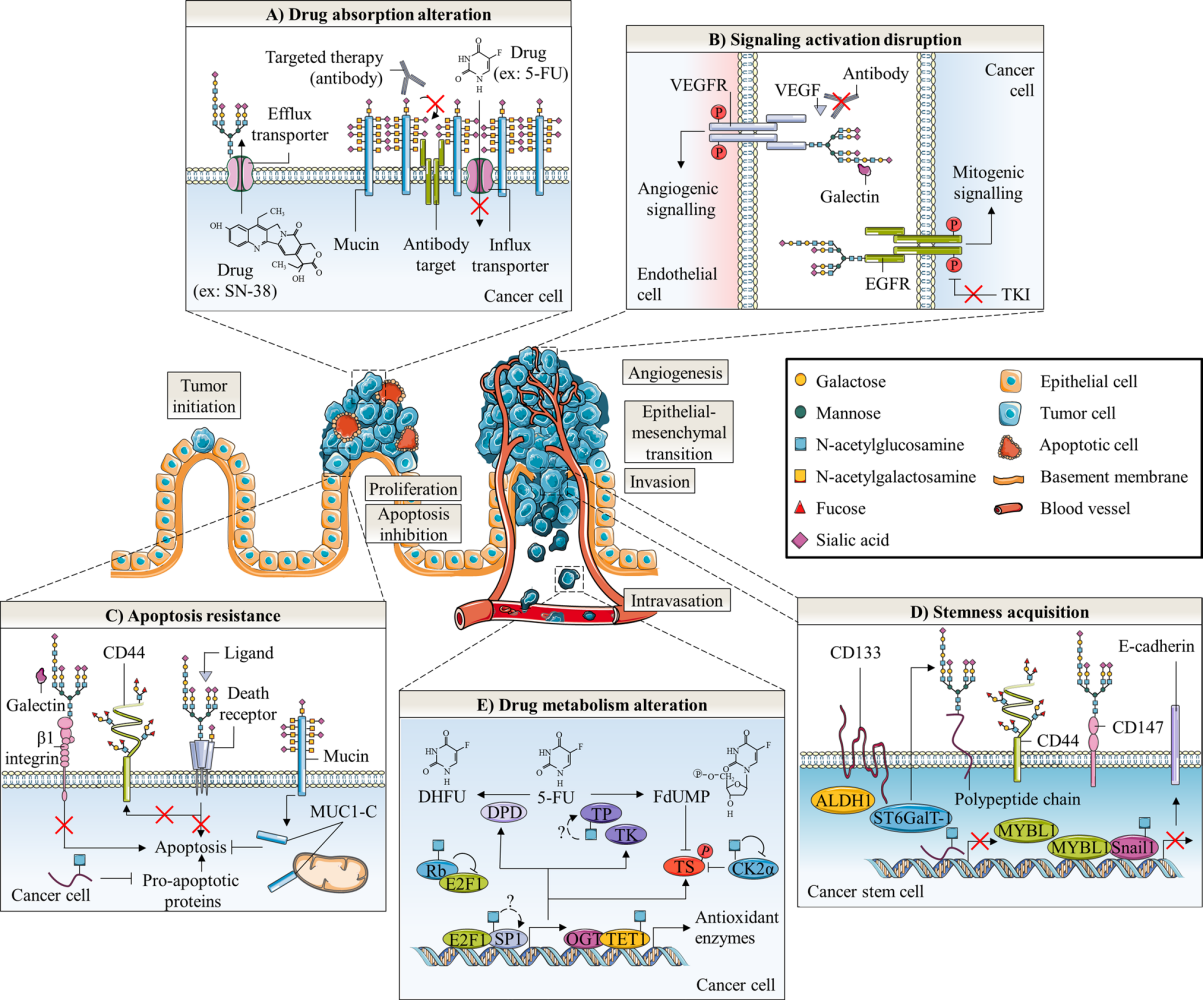
Glycosylation roles in anticancer therapy resistance of colorectal cancer Abnormal glycosylation associated with colorectal cancer is involved in cancer emergence and progression as well as in different anticancer therapy resistance mechanisms. These later include altered drug absorption (**A**), signaling activation disruption (**B**), apoptosis resistance (**C**), cell stemness acquisition (**D**) and altered drug metabolism (**E**). A) Mucins could generate steric hindrance, mask surface antigen of targeted therapy and decrease absorption of chemotherapy. In contrast, *N*-glycosylation can stabilize transporters responsible for efflux of chemotherapy drugs. B) On endothelial cells, aberrant β1,6-GlcNAc bearing-*N*-glycans of VEGFR could promote its interaction with galectins and activate angiogenic signaling in the absence of ligand resulting in decreased efficacy of anti-VEGF treatment. In tumor cells, the presence of α2,6-sialylated terminal structure on EGFR could also decreased activity of the receptor and efficacy of TKI. C) Abnormal glycosylation of β1 integrin prevents binding and pro-apoptotic activity of Gal. Both altered *N*- and *O*-glycosylation of cell death receptors TNFR1, FasR, DR4 and DR5 inhibit induction of apoptosis. Fucosylation of Lewis antigen structures on CD44 could protect this CSC biomarker from proteolytic cleavage occurring in early steps of apoptosis. Truncated *O*-glycosylation of mucins promotes their intracellular accumulation and outer mitochondrial localization of MUC1-C could affect activation of the intrinsic apoptotic pathway. *O*-GlcNAcylation regulates expression of pro-apoptotic proteins. Altered *O*-GlcNAcylation could therefore induce resistance to apoptosis. D) The EMT process is supposed to generate CSC which have a slow proliferation rate and are thus less sensitive to chemotherapies. CRC CSC biomarkers CD44 and CD147 can be glycosylated. Expression of ST6GalT-1 is correlated with expression of ALDH1 and CD133 stem cell markers and proportion of CSC. The CRC CSC compartment could be regulated by the *O*-GlcNAcylation mediated-epigenetic down-regulation of MYBL1, a transcription activator of E-cadherin. *O*-GlcNAcylation also stabilizes the E-cadherin suppressor Snail1, essential for the loss of adherens junctions associated with EMT. E) *O*-GlcNAcylation may take fundamental part in regulation of 5-FU metabolism in colon cancer cells. OGT interacts with TET1 on promoter regions to activate transcription of antioxidant enzymes such as Nrf2. *O*-GlcNAcylation regulates both transcription factors Sp1 and E2F-1, which regulate a myriad of genes involved in nucleotide synthesis including enzymes involved in 5-FU catabolism and anabolism such as DHFR, TK and TS. E2F-1 is sequestered and inactived by binding to hypo-phosphorylated and *O*-GlcNAcylated Rb during early G1 phase of cell cycle. On the other hand, *O*-GlcNAcylation of E2F-1 was found to regulate its stability and its transcriptional activity but the specific biological effect in colon cancer cells has not been reported yet. TP was also found to be *O*-GlcNAcylated in breast tumors but the functional role of this glycosylation has not been studied. Finally, a Yin-Yang relation between phosphorylation and *O*-GlcNAcylation is expected to affect activity of TS, the target of active metabolite FdUMP. *O*-GlcNAcylation at Ser347 appears to decrease Thr344 phosphorylation and stability of CK2α. CK2α is responsible for the phosphorylation at Ser124 and the decrease of catalytic activity of TS.

The use of glycosylation inhibitors demonstrated the importance of *N*-glycosylation in chemoresistance mechanisms. First, swainsonine, a potent inhibitor of α-mannosidase 2 that is essential for the production of complex-type *N*-glycans, increased 5-FU sensitivity in all induced-resistant cell lines established from colon 26, a mouse cancer colorectal cell line, but not in its parental one [40]. The authors observed that *N*-glycan profiles of both the resistant and parental cells were changed by swainsonine treatment and showed that alterations in the *N*-glycan structure affected mechanisms of 5-FU resistance by up-regulating thymidylate synthetase (TS) expression and down-regulating orotic acid phosphoribosyltransferase (OPRT) expression at the transcriptional level (Figure 3). However, the underlying mechanism correlating *N*-glycan structures profile and 5-FU metabolizing-enzymes expression is not yet elucidated. Drug-metabolizing enzymes play an important role in reducing the intracellular accumulation of drugs. Their expression can therefore either potentiate or reduce the drugs toxicity, and variations in both the anabolic and catabolic pathways can lead to drug resistance. Increased expression of TS, a key enzyme in pyrimidine metabolism and the major target of 5-FU, and decreased expression of the anabolic enzyme OPRT have already been associated with 5-FU resistance [41]. Secondly, ATP-binding cassette (ABC) sub-family G member 2 (ABCG2) is an *N*-linked glycosylated transporter responsible for efflux of the active metabolite of irinotecan 7-ethyl-10-hydroxycamptothecin (SN-38) (Figure 3). Overexpression of this efflux pump in cancer cells gives them also the ability to reduce chemotherapy intracellular concentration [41, 42]. Disruption of the early steps of the dolichol pyrophosphate precursor biosynthesis by treatment of Flp-ln-293 embryonic kidney cells with tunicamycin decreases stability of ABCG2 and reduces cell resistance to SN-38 [43] (Figure 4A). In the same way, Santos et al. demonstrated an increased antitumor activity by combined administration of swainsonine and cisplatin *in vivo* [44]. Authors suggested that swainsonine may interfere with ABCB1 activity, a broad-spectrum multidrug efflux pump whose activity is dependent on *N*-glycosylation. Based on the aforementioned data, *N*-glycosylation can alter intracellular drug accumulation by reducing drug anabolism and increasing drug export. Targeting *N*-glycosylation may improve cancer chemotherapy and reduce drug resistance.

#### Beta1,6-GlcNAc bearing *N*-glycans implication in drug resistance

Increased expression of β1,6-GlcNAc bearing *N*-glycans is a frequent cancer-associated modification due to abnormal expression of GnT-5. In the Golgi apparatus, GnT-5 catalyzes the transfer of GlcNAc to the trimannosyl core of *N*-glycan to produce tri or tetra-antennary complex glycans (Figure 1). GnT-5 expression is increased in different cancers [45, 46] including colon cancer [29] (Figure 2). In CRC, GnT-5 expression is associated with metastasis likely through glycosylation of the tissue inhibitor of metalloproteinase-1 (TIMP-1). Aberrant *N*-glycosylation of TIMP-1 affects its binding properties with matrix metalloproteinases (MMP) increasing cancer cell invasiveness [47].

The contribution of β1,6-branched *N*-glycans in cancer angiogenesis was reinforced by the observation that vasculature of anti-VEGF resistant mice tumors presented high levels of β1,6-GlcNAc branched *N*-glycans. In this model, GnT-5 knockdown increased efficacy of anti-VEGF treatment by modulating the interaction of some β-galactoside-binding lectins galectins with cell surface glycoproteins. Galectin-1 (Gal-1) is able to bind complex-type *N*-glycans on endothelial cell VEGF receptor 2 (VEGFR-2) and to activate an angiogenic signaling pathway in the absence of VEGF-A ligand. In fact, binding of Gal-1 resulted in VEGFR-2 clustering into membrane microdomains and increased surface retention. It was suggested that loss of β1,6-GlcNAc *N*-glycan branching prevents Gal-1 binding to endothelial cells [48] (Figure 4B; Table 1). In this way, extrinsic properties of tumor-associated microenvironment can influence directly the drug response in cancer. Activation of the angiogenic pathway by the GnT-5 mediated *N*-glycosylation of VEGFR-2 in the absence of ligand binding could be responsible, in part, of failure of anti-VEGF treatment. To circumvent this resistance, Croci et al. proposed targeting glycosylation-dependent galectin-receptor interactions and validated therapeutic efficiency of an anti-Gal-1 neutralizing monoclonal antibody [48]. Alternative therapeutic strategies targeting galectin-glycan interactions include β-galactose ligands, talose-based ligands and mimicking glycans [49]. In the other hand, we can also consider blocking the VEGF-VEGFR-2 signaling at a downstream level for example with receptor tyrosine kinases (RTK) inhibitors (TKI). A second-generation of these agents with higher specificity for VEGFR inhibition and favorable toxicity profiles are currently under clinical evaluation in metastatic CRC [50].

**Table 1:** Glycosylations involved in anti-cancer drug response of colorectal cancer

Types of glycosylation	Glycosyl-transferases involved	Targets	Functional roles	Drug responses	Cell types/tissues	References
β1,6 branching of *N*-glycans	GnT-5	VEGFR-2	Promotes pro-angiogenic Gal-1 binding	Decreases anti-VEGF antibody sensibility	HUVEC1 cells and transgenic mice models	[48]
Poly-LacNAc chain on *N*-glycans	β3GnT-8	-	-	Decreases 5-FU sensitivity	SW6202 cells	[53]
α2,6-linked sialylation of *N*-glycans	ST6GalT-1	VEGFR-2	Prevents pro-angiogenic Gal-1 binding	Increases anti-VEGF antibody sensitivity	HUVEC1 cells and transgenic mice models	[48]
		β1 integrin	Prevents pro-apoptotic Gal-3 binding	Decreases Gal-3 sensitivity	SW482 cells	[73]
		FasR	Prevents formation of active DISC	Decreases FasL sensitivity	HD32 and SW482 cells	[77]
		EGFR	Decreases EGF-mediated phosphorylation and activation	Decreases gefitinib sensitivity	SW4802, HT-292, HCT-1162 and SW482 cells	[67]
		-	Increases cancer stem cells population	Decreases irinotecan sensitivity	HD32 and SW9482 cells	[69]
Mucin-type *O*-glycosylation	ppGalNAcT-3	-	-	Increases TRAIL sensitivity	DLD-12 and C1702 cells	[113]
	ppGalNAcT-14	-	-	“TT” genotype negatively correlated with oxaliplatin sensitivity	Patients with stage III colorectal cancer (CRC)	[115]
Lewis blood group antigen structures	FucT-1/FucT-2	-	-	Decreases 5-FU sensitivity	REG3 and PRO3 cells	[122]
					REG3 and PRO3 cells	[123]
					DLD-12 cells	[124]
	FucT-3	-	-	Expression of FUT-3 positively correlates with TRAIL sensitivity	CRC cell lines2 panel	[113]
	FucT-6	-	Increases DISC activation of capase-8	Increases TRAIL sensitivity	DLD-12 and C1702 cells	[113]
	-	-	-	Expression of sLex negatively correlates with irinotecan/5-FU/leucovorin therapy sensitivity	Patients with advanced or recurrent CRC	[131]
*O*-GlcNAcylation	OGT	-	Interacts with TET1 and SET1/COMPASS complex on promoter region of Nrf2	Decreases 5-FU sensitivity	SNUC52 cells	[161]
		-	-	OGT expression negatively correlates with FdUMP sensitivity	NCI-602 cells	[165]

- : not defined; 1: human umbilical vein endothelial cells; 2: human colorectal carcinoma cells; 3: rat colorectal carcinoma cells.

#### Poly-LacNAc chain bearing *N*-glycans implication in drug resistance

Among the β3GnT family, β3GnT-8 is the most recently identified enzyme involved in the biosynthesis of poly-LacNAc chain on tetra-antennary *N*-glycans [30] (Figure 1). The β3GnT-8 transcript level is almost undetectable in normal colon tissues while it is increased in cancer tissues [30] (Figure 2). The β3GnT-8 expression is also correlated with metastatic potential of CRC cells notably by targeting cluster of differentiation 147 (CD147), also known as extracellular matrix metalloproteinase inducer (EMMPRIN) [51]. High *N*-glycosylated CD147 (HG-CD147), the active glycoform, is enriched on cancer cell surface and promotes production of secreted MMP in tumor cells themselves and in adjacent stromal cells [52].

Ni et al. showed that β3GnT-8 overexpression and silencing respectively increases and decreases levels of HG-CD147 in LoVo metastatic colon cancer cell line revealing that HG-CD147 glycans consists of β1,6-branched poly-LacNAc chains [51]. In parallel, β3GnT-8 is overexpressed in 5-FU resistant colon cell line SW620 compared to parental sensitive cells and knockdown of β3GnT-8 resensitizes cells to 5-FU-mediated apoptosis. However, the underlying mechanism leading to 5-FU resistance is unknown [53]. Recent findings suggest that glycosylated CD147 plays an essential role in multidrug resistance mechanism. CD147 is associated with CRC stem cells (CSC) [54], a minor cell population characterized by a slow rate of proliferation, an undifferentiated phenotype, a self-renewal potential and the capacity to generate differentiated progeny [55]. The longevity of CSC renders them more vulnerable to accumulate DNA damages and epigenetic alterations that may promote the proliferation of heterogeneous and aggressive cell phenotypes [55, 56]. Furthermore, some CSC can be found in hypoxic tumor niches distant from functional blood vessels which favor the maintenance of their undifferentiated state and exposed them to suboptimal drug concentrations [57]. In this sense, CSC appear to be more resistant towards chemotherapy than more proliferative progenitors coexisting within the tumor. It was shown that CD147 promotes epithelial-mesenchymal transition (EMT) [58], a trans-differentiation process by which epithelial-polarized cells acquire a mesenchymal phenotype and generate some cancer cells with stem-like cell properties [59, 60] (Figure 4D). Silencing CD147 sensitized cells to chemotherapeutic reagents including 5-FU [58], oxaliplatin [61] gemcitabine, cisplatin and docetaxel [54]. We may hypothesize that stimulation of the EMT by HG-CD147 leads to the formation of a pool of slowly-proliferative chemoresistant CSC. The therapy actually envisaged to inhibit this resistance mechanism is to specifically recognize and eliminate CSC [62, 63]. Thus, HG-CD147 may constitute a potential chemoresistance biomarker useful for identifying and therapeutically targeting CSC.

#### N-glycosylation alterations in colorectal cancer: a cause of drug resistance

Increased terminal sialylation of branched *N*-glycans is a common feature of cancer. ST6GalT-1 is a Golgi enzyme that catalyzes the transfer of the sialic acid Neu5Ac to the terminal galactose of *N*-glycans (Figure 1). ST6GalT-1 expression and global α2,6-sialylation are up-regulated in CRC [64] (Figure 2) and in many other cancers [65]. Ras oncogene that transcriptionally drives ST6GalT-1 expression is mutated in 50% of CRC leading to the glycosyltransferase expression increase [66]. ST6GalT-1 expression is positively correlated with metastatic potential of colon cancer cells [67, 68], CSC markers expression [69] and poor prognostic [70]. ST6GalT-1 enhances migration and invasion properties of tumor cells. On the one hand, the enzyme activates the phosphoinositide 3-kinase (PI3K)/Akt mitogenic signaling pathway *in vitro* [71] and *in vivo* [72] by targeting integrins and potentially some RTK. On the other hand, forced α2,6-sialylation of β1 integrin subunit enhances its activity and subsequent cell migration by improving extracellular matrix (ECM)/cytoskeleton interactions [68].

Accumulating studies showed that ST6GalT-1 displays anti-apoptotic activities. First, it has been reported that ST6GalT-1 regulates apoptosis signaling in response to galectins [73]. In this way, overexpression of ST6GalT-1 and α2,6-sialylation of β1 integrins prevents galectin-3 (Gal-3) binding and pro-apoptotic activity in SW48 colon cancer cells (Figure 4C). Intriguingly, mice tumor vessels exhibiting high levels of α2,6-sialylation were more sensitive to anti-VEGF whereas ST6GalT-1 knockout mice lacking this modification resisted to anti-VEGF therapy [48]. Contrary to β1,6-branched *N*-glycans, loss of α2,6-sialylation may activate VEGF-like pathway by unmasking Gal-1-specific binding sites on ligands such as VEGFR-2 [48] (Table 1). Next to the galectin-mediating pathway, α2,6-sialylation of EGFR decreased its EGF-mediated tyrosine phosphorylation activity and promotes resistance to gefitinib, an EGFR-targeted TKI (Figure 4B; Table 1). The cytotoxic effect of gefitinib is respectively decreased and increased in CRC cell lines overexpressing or deficient in ST6GalT-1 [67]. Alpha2,6-sialylation of EGFR could prevent its dimerization and activation as it was shown that sialidase increased EGFR dimer formation upon EGF treatment in lung cancer cells [74]. Interestingly, EGFR gene copy number is strongly positively-associated with sensitivity to EGFR TKI in CRC [75, 76]. Together, these results indicate that active EGFR is necessary to effective response to TKI. Due to heterogeneous scoring systems and technical obstacles, estimation of EGFR gene copy number as EGFR TKI predictive biomarker is still unpractical in clinical practice [76]. Alpha2,6-sialylation of EGFR could be envisaged as another potential predictive biomarker. In addition, α2,6-sialylation could inhibit apoptosis initiated by the Fas ligand (FasL) binding to Fas cell death receptor (FasR) [77]. In fact, ST6GalT-1 mediated α2,6-sialylation of FasR prevents the formation of active death inducing signaling complex (DISC) by blocking the binding of the Fas-associated adaptor protein (FADD) to the FasR death domain and inhibits the internalization of stimulated FasR necessary for induction of apoptosis (Figure 4C; Table 1). It was similarly reported that ST6GalT-1-mediated sialylation of the tumor necrosis factor receptor 1 (TNFR1) protects macrophages against TNF-α-induced apoptosis [78]. Cytotoxic effects of anti-cancer drugs are generally mediated by the activation of intrinsic and extrinsic pro-apoptotic cascades in response to DNA damages or other cellular injuries [79]. Anti-CRC drugs activate the extrinsic apoptotic pathway via up-regulation of expression and/or activation of death-receptors and downstream actors [80]. Evasion of apoptosis mediated by increased expression of anti-apoptotic proteins and decreased expression of pro-apoptotic proteins contributes to drug resistance [79]. Some targeted agents are currently evaluated to restore the impaired apoptotic signals in CRC. These include death receptor ligands or agonists, B-cell lymphoma 2 (Bcl-2) antagonists, Bcl-2 homology 3 (BH3) mimetics or inhibitors of apoptosis (IAP) antagonists in monotherapy or in combination with standard chemotherapy regimens [80, 81]. Detection of aberrant *N*-glycosylation on inactive death receptors could allow choosing an appropriate therapeutic strategy by favoring targeted therapies against downstream effectors in the extrinsic apoptotic pathway such as IAP. Finally, ST6GalT-1 expression is restricted to stem cell compartment at the base of colonic crypts in normal colon tissue and correlated with aldehyde dehydrogenase 1 (ALDH1) stem cell marker [69]. In colon carcinoma cell lines, ST6GalT-1 expression also correlates with CD133/ALDH1 positive CSC [69]. Conversely, knockdown of ST6GalT-1 decreases the proportion of CSC. Irinotecan resistant cells show an increase in expression and activity of ST6GalT-1 and a greater proportion of CSC compared to parental sensitive cells (Figure 4D). In this study, the authors regard ST6GalT-1 as a CSC potential marker.

### Mucin-type *O*-glycosylation profile alterations in colorectal cancer: a barrier to drug therapy

#### Truncated mucin-type *O*-glycosylation in CRC

High density of truncated mucins is another common tumor-associated carbohydrate modification. Compared to normal colon mucins which exhibit the four core structures (Figure 1) and mainly core-3 based *O*-glycans [82], during malignant transformation mucins present a dramatic decrease of core 3 and core 4 and an increase of T antigen. These modifications result from the deregulation of the glycosyltransferases involved in the building of the different structures [31–33] (Figure 2). The Sda blood group epitope bound to *O*-glycans core-3 is mostly expressed in healthy colorectal tissue but not in CRC cells. Accordingly, the expression of β1,4-N-acetylgalactosaminyltransferase 2 (β4GalNAcT-2) which synthetizes Sda epitope is decreased in CRC [83]. While T antigen is considered as an oncodevelopmental cancer-associated antigen and is overexpressed early during colon tumorigenesis, Tn and sTn antigens overexpression correlates with advanced and poorly differentiated colon carcinomas and is associated with a poor clinical outcome [84]. Moreover, mucin-type C2GnT (C2GnT-M) expression, encoded by GCNT3 gene, is down-regulated in early stage colon cancer in comparison to normal colon tissues [85]. Its overexpression inhibits cell growth, adhesion, migration and invasion and induces apoptotic cell death [86]. Recently, González-Vallinas et al. revealed that 5-FU induces a significant dose-dependent overexpression of GCNT3 in sensitive parental SW620 colon cancer cell line but not in resistant one reinforcing the role of C2GnT-M in inhibition of tumor progression [85].

#### Drug resistance associated with dysregulation of expression, glycosylation and subcellular localization of mucins

Normal colon expresses highly MUC2, weakly MUC1, MUC3 and MUC4 and rarely MUC5AC, MUC5B and MUC6 [87–89]. Compared to normal tissues, hypoglycosylated MUC1 is overexpressed all over the surface of cancer cells and correlates with aggressiveness of carcinomas [90] whereas a decrease of MUC2 expression was described in colorectal adenocarcinoma but not in mucinous carcinomas [91, 92]. Recent studies demonstrate a relationship between the CRC classification and mucins expression pattern. According to genetic pathways involved, CRC follow the chromosomal instability (CIN) pathway with loss of heterozygosity (LOH) of tumor suppressor genes (such as adenomatous polyposis coli (APC) and tumor protein 53 (TP53)) and activation of proto-oncogenes (such as V-Ki-ras2 Kirsten rat sarcoma viral oncogene homolog (KRAS)) or the microsatellite instability (MSI) pathway with frequent aberrations in the DNA mismatch repair (MMR) machinery [93]. A strong correlation was reported between a mucinous phenotype (overexpression of MUC2, MUC5AC and MUC6) and sporadic MSI-high tumors characterized by V-Raf murine sarcoma viral oncogene homolog B (BRAF) mutation in which valine (Val, V) is substituted for glutamic acid (Glu, E) at residue 600 (p.V600E) and an extensive DNA methylation pattern also known as the CpG island methylator phenotype (CIMP) [94–96]. Increased secreted mucins expression can be attributed to promoters hypomethylation [95] and/or activation of the EGFR-RAS-RAF pathway [97].

Lesuffleur et al. showed that, compared to parental sensitive HT-29 cells, the 5-FU resistant mucus-secreting subclone overexpressed MUC1, MUC2 and MUC4 and underexpressed MUC3 at the transcript level [98]. Interestingly, cells resistant to methotrexate exhibit a different profile of mucins expression [98]. The increased level of MUC2 transcript in 5-FU resistant HT-29 cells has been further confirmed by [99]. Patients suffering mucinous adenocarcinoma and receiving 5-FU show a diminished clinical response compared to those with nonmucinous tumors; this discrepancy likely results from a significant higher expression of TYMS gene encoding TS [100] (Figure 3). Mucinous tumors overexpress also glutathione S-transferase pi 1 (GSTP1) gene [100] encoding enzyme involved in the detoxification of platinum agents by glutathione (GSH) conjugation (Figure 3) expecting a weak response to oxaliplatin-treatment. CRC classified as MSI tumors showed higher mutation frequency and expression of TYMS than microsatellite-stable (MSS) tumors [101, 102] suggesting a link between MSI status, mucinous profile and chemoresistance. The overexpression of *O*-glycosylated membrane-bound mucins can generate steric hindrance and mask surface antigens or decrease absorption of chemotherapy [103] (Figure 4A). In normal colon, there is no or a faint detection by monoclonal antibodies of the polypeptidic backbone of MUC1 which is masked by high density of long and complex mucin-type *O*-glycan chains. However, in CRC cells, MUC1 exhibits much shorter carbohydrate side-chains such as Tn and sTn allowing its immunodetection [104]. This aberrant glycosylation leads to MUC1 clathrin-mediated endocytosis [105]. It is also thought that MUC1 hypoglycosylation unmasks its polypeptidic core promoting MUC1 amino-terminal (N-ter) (MUC1-N) subunit proteolytic cleavage and release by extracellular proteases [105, 106]. The internalization of MUC1 carboxy-terminal (C-ter) (MUC1-C) subunit may initiate oncogenic signaling [105, 106]. Moreover, an increase in negatively charged sialic acid residues on MUC1 may contribute to metastasis progression by interfering with cell-cell adhesion. MUC1 confers resistance to reactive oxygen species (ROS)-induced apoptosis [107], hypoxia [108] and chemotherapeutic drugs [109]. Outer mitochondrial membrane localization of MUC1-C downregulates cisplatin-induced release of mitochondrial pro-apoptotic factors, activation of caspase-3 and of the intrinsic apoptotic pathway *in vitro* and *in vivo*, in part, by stabilization of the mitochondrial membrane potential [109] (Figure 4C). MUC13 is also overexpressed in the cytoplasmic compartment of CRC carcinomas [110]. Its silencing sensitizes colon cancer cell lines to different DNA damaging agents including 5-FU, oxaliplatin, vincristine and doxorubicin *in vitro*, and suppresses tumor growth *in vivo* by decreasing subsequent activation of the nuclear factor-kappa B (NF-κB) survival pathway [110]. However, the *O*-glycosylation profile of these mucins in CRC tumors has not been studied yet. Like for MUC1, an aberrant *O*-glycosylation of MUC13 may affect its subcellular localization and favor to activate oncogenic pathways. In this sense, it was speculated that overexpression of ppGalNAcT-14 contributes to ovarian carcinoma migration through truncated *O*-glycosylation of its substrates including MUC13 [111].

#### ppGalNAcTs: key enzymes involved in multidrug resistance

Treatment of CRC cells with benzyl-2-acetamido-2-deoxy-α-D-galactopyranoside (benzyl-α-GalNAc), a structural analog of α-GalNAc linked to serine or threonine residue which competitively inhibits core 1 *O*-glycans extension, or benzyl-α-GalNAc-derived oligosaccharides has been shown to induce apoptosis [112] and reduce sensitivity to TNF-related apoptosis-inducing ligand (TRAIL) [113]. While knockdown of ppGalNAcT-3 decreases sensitivity of CRC cells to TRAIL (Table 1), expression of ppGalNAcT-14 correlates with responsiveness in pancreatic carcinoma, non-small-cell lung carcinoma (NSCLC) and melanoma. Notably, it was shown that ppGalNAcT-14 catalyzes the *O*-glycosylation of death receptors 4 and 5 (DR4 and DR5) in PSN-1 pancreatic cancer cells leading to TRAIL-induced receptors clustering and apoptosis. This *O*-glycosylation may stabilize death receptors in the plasma membrane or enhance their ligand-binding properties. In hepatocellular carcinoma, the single nucleotide polymorphism (SNP) marker rs9679162 “TT” genotype located in the ppGalNAcT-14 gene is associated with a favorable outcome in 5-FU, mitoxantrone and cisplatin combination chemotherapy treated patients [114]. It is suggested that ppGalNAcT-14 enhances sensitivity to therapy linked to TRAIL-mediated apoptosis through modulation of DR4 and/or DR5 *O*-glycosylation. However, biological effect of this genotype on the expression or activity of ppGalNAcT-14 is not yet known. In contrast, ppGalNAcT-14 “TT” genotype is correlated with poor outcome and tumor invasion in advanced CRC patients treated with oxaliplatin-based chemotherapy [115] (Table 1). The prognostic value of ppGalNAcT-14 “TT” genotype seems to be tissue and/or chemotherapy-dependent reflecting likely the involvement of different target glycoproteins.

#### Altered fucosylation/sialylation profile of Lewis antigens associated with drug resistance in colorectal cancer

Lewis carbohydrate determinants reside in glycolipids and glycoproteins at the surface of most epithelia (Figure 1) and are overexpressed in many carcinomas [12] including colon cancer [116]. Normal colon mucosa expresses complex glycans such as disialyl-Lea and sialyl-6-sulfo-Lex at higher levels whereas the expression of sLea and sLex is quite low [117, 118]. The expression of these structures is inverted in CRC where increase of sLea and sLex amounts is associated with advanced tumors, metastasis occurrence and poor prognosis [119, 120]. The interaction of these antigens with the adhesion molecule E-selectin in cytokine-activated endothelial cells has been proposed to play a role in the invasion and metastasis of cancer cells [121]. Leb and Ley are considered as typical oncofetal antigens in CRC (Figure 2) due to increased activity of α2FucT [35, 36].

Several studies describe a link between expression of fucosylated antigens and sensitivity of cancer cells to apoptosis. First, Goupille et al. showed that ectopic expression of rat α2FucT FTA or FTB cDNA, respectively homologous to human FucT-1 and FucT-2, increased levels of α1,2-fucosylated structures, tumorigenicity and resistance to apoptosis in spontaneously regressing REG rat colon adenocarcinoma cell clone [122]. Inversely, transfection of spontaneously tumorigenic PRO cells forming progressive tumors and presenting surface α1,2-fucosylated antigens with FTA antisense cDNA sensitized PRO cells to cell death (Table 1). An interplay between expression of H2 structures, the synthesis intermediates for Ley and Lex antigens (Figure 1), and cell response to 5-FU has also been observed [123]. In one hand, 5-FU treatment induces an increase of both α2FucT activity and H2-type structures *in vitro* and *in vivo*. On the other hand, FTA knockdown and overexpression respectively increased and decreased cells sensitivity to 5-FU (Table 1). The involvement of α1,2-fucosylated antigens in the resistance to 5-FU treatment was further confirmed [124] (Table 1). One of the major identified glycoproteins carrying the H2-type determinant is the CD44 adhesion molecule, a putative cancer stem cell marker [55, 123]. Normal colon cells express the standard form of CD44 (CD44s) whereas cancer cells may also express CD44 variants (CD44v) bearing supplementary domains underlying new oncogenic functions. In colon cancer, expression of CD44s is linked to EMT [125] and expression of CD44v3 and v6 is associated with metastatic phenotype [126]. Depletion of the CD44s by proteolytic cleavage in early steps of Fas-triggered apoptosis contributes to loss of cell-cell and cell-matrix anchorage [127]. Moreover, resistant HT-29 and HCT-116 colon cancer cells to 5-FU, oxaliplatin and SN-38 showed a highest expression of CD44s compared to sensitive parental cells [128]. Cordel and collaborators speculated that increased expression of α2FucT could participate in acquired-drug resistance by fucosylating CD44 and protecting it from proteolytic cleavage or strengthening cell adhesion [123] (Figure 4C). Yazawa et al. also found that 5-FU resistant DLD-1 colon carcinoma human cells have α2FucT and α3FucT higher activities and less of α4FucT activity [124]. Treatment of these cells with the α2FucT substrate phenyl β-galactoside decreased immunodetection of Ley, Leb and H2-type structures with YB-2 anti-fucosylated antibody and increased sensitivity to 5-FU suggesting the involvement of Leb and/or Ley antigens in the resistance mechanism. In parallel, expression of α3/4FucT FucT-3 and α3FucT FucT-6 correlates with TRAIL sensitivity and it was shown that FucT-6 knockdown reduced DISC activation of pro-apoptotic caspase-8 in DLD-1 cells [113] (Table 1). Moreover, it was demonstrated that interferon gamma (IFNγ) and anti-Fas antibody treatment-mediated apoptosis induce an increase of transcript level of FucT-4, of α2FucT and α3FucT activities, and of Lex and Ley antigens at the cell surface [129]. Contradictory results showed that expression of Lex and Ley antigens was significantly diminished in colon cells treated by anti-Fas apoptosis inducers [130]. Finally, Yanagisawa et al. revealed that responder CRC patients to modified irinotecan/5-FU/LV therapy harbor significant lower expression of sLex on core 2 branched mucin-type *O*-glycans [131] (Table 1). The nature of the fucosylated-antigens expressed at the cell surface of colorectal cancer cells might impact differently the mechanism of drug-induced apoptosis.

### *O*-GlcNAcylation and drug resistance in cancer: TERRA INCOGNITA

*O*-GlcNAcylation is a recently discovered glycosylation whose role in cancer has been well studied in contrast to its involvement in drug response. In the following section, we will give a brief overview of what is known about *O*-GlcNAcylation in cancer before discussing the link with drug resistance.

#### Upregulation of OGT and *O*-GlcNAcylation: impact in cancer

An increased *O*-GlcNAcylation has been observed in many cancers [28, 132] including colon [133]. In colon cancer cells, the increased level of *O*-GlcNAcylation correlates with an overexpression of nucleocytoplasmic OGT [134, 135]. Contradictory findings are published concerning OGA expression. While Mi et al. did not report any change in OGA expression between colon cancer cells and normal adjacent tissues [133], Phueaouan et al. [136] and us [134, 135] observed instead an overexpression of OGA *in vitro* and *in vivo*. A fine cross-regulation between OGT and OGA expression was reported by different groups but the underlying mechanism by which each enzyme regulates the expression of the other is not fully understood. *O*-GlcNAcylation is an important regulator of cell proliferation, survival and migration especially through its interplay with phosphorylation [137]. *O*-GlcNAcylation targets and regulates the activity or the fate of oncoproteins such as β-catenin [134, 138–140], c-Myc [141, 142] and forkhead box protein M1 (FoxM1) [143], and of tumor suppressors like p53 [144] and the retinoblastoma protein (Rb) [145]. *O*-GlcNAcylation is known to promote EMT in CRC. Beta-catenin is mutated in 10% of CRC [146] and its *O*-GlcNAcylation is suggested to participate in colorectal tumorigenesis in a context of glucose metabolism deregulation [147]. An elevation of *O*-GlcNAcylation in colon cancer cells reduced phosphorylation and ubiquitination of β-catenin increasing therefore its stability, reducing its localization to adherens junctions and enhancing its nuclear accumulation and activation [134, 138, 139]. Snail1 is a transcriptional repressor of epithelial cadherin (E-cadherin) whose expression is associated with EMT. Snail1 is also stabilized by *O*-GlcNAcylation in colon cancer cells [148]. Reduced levels of *O*-GlcNAcylation diminished the colorectal CSC compartment *in vivo* by overexpression of the epigenetic regulation of MYB proto-oncogene like 1 (MYBL1), a transcriptional activator of E-cadherin [149] (Figure 4D). The key role of *O*-GlcNAcylation of other EMT actors has also been well highlighted [150, 151]. The comprehension of the mechanisms underlying the role of *O*-GlcNAcylation in cell apoptosis remains at its beginnings. However, some data give evidence that OGA and OGT isoforms play different roles in cell death signaling [152]. Thus, very few studies have examined specifically the role of *O*-GlcNAcylation in regulation of CRC cell death signaling. Elements of answer may be suggested according to studies in other cell types. First, an increase of global *O*-GlcNAcylation level was observed in Cos-7 green monkey kidney cells in response to cellular stresses strengthening the idea that *O*-GlcNAcylation enhances survival by regulating several cell death pathways [153, 154]. Reducing *O*-GlcNAcylation in pancreatic ductal adenocarcinoma (PDAC) cell lines inhibits constitutive NF-кB activity and induces apoptosis [155] while, increased *O*-GlcNAcylation protects BxPC-3 pancreatic adenocarcinoma cells against death [155]. Interestingly, OGT knockdown did not trigger apoptosis in non-transformed pancreatic duct epithelial (HPDE) cells. Similarly, OGT knockdown induces apoptosis in MDA-MB-231 breast cancer cells but not in MCF-10A immortalized mammary epithelial cells through hypoxia-inducible factor 1-α (HIF-1α) degradation, decrease of glycolysis and ER stress pathway activation [156]. Yang et al. showed that p53 - the guardian of the genome – is stabilized by *O*-GlcNAcylation in MCF-7 breast carcinoma cells [144]. Fardini et al. suggested a dual impact of *O*-GlcNAcylation on p53 depending upon the physiopathological context. The glycosylation promotes tumor suppressor activity of wild-type p53 and amplifies the pro-oncogenic activity of gain-of-function mutant form of p53 [28]. TP53 mutation status has been correlated to CRC response to drug therapy. In particular, the non-synonymous exonic SNP rs1042522 resulting in Pro substitution at codon 72 (P72) enhances the function of the protein. In fact, p53-P72 mutant has an increased capability to DNA binding and transcription activation of target genes. Interestingly, CRC patients carrying this gain-of-function mutation benefited most from 5-FU-based chemotherapies [157]. Recently, Gokare et al. demonstrated that p53-P72 repressed expression of dihydropyrimidine dehydrogenase (DPD), the rate-limiting enzyme in the 5-FU catabolism (Figure 3), following TS inhibition [158]. Thus, *O*-GlcNAcylation might play a role in 5-FU sensitivity by modulating activity of p53 wild-type and mutants.

The increasing number of evidences pointing out the crucial role of *O*-GlcNAcylation in the biological processes underlying tumorigenesis led to consider and then analyze this glycosylation and its related enzymes as potential actors of drug resistance.

#### *O*-GlcNAcylation and chemotherapy sensitivity

Several studies have correlated expression of OGT and chemotherapy sensitivity. OGT silencing increases the sensitivity to 5-FU of BCG-823 gastric cancer cell line by enhancing expression of p53 upregulated modulator of apoptosis (PUMA) and caspase-3 pro-apoptotic proteins [159]. In this sense, Pepe et al. showed in HepG2 hepatoblastoma cells that OGT stabilizes the transcriptional complex β-catenin/upstream stimulatory factor 1 (USF1) at the promoter of miR-483-3p, a microRNA responsible for transcription downregulation of PUMA [160]. Treatment with 2-deoxy-D-glucose (2-DG), a glucose-mimic inhibitor of glycolysis, reduces miR-483-3p expression and increases sensitivity to 5-FU-induced apoptosis. In 5-FU resistant SNUC5 colon cancer cells, OGT is overexpressed and interacts strongly with oxidative stress-activated ten-eleven translocation methylcytosine dioxygenase 1 (TET1) and histone H3 lysine 4 (H3K4) methyltransferase SET1/COMPASS (complex proteins associated with SET1) complex involved in the activation of gene expression [161]. *O*-GlcNAcylation is known to modulate recruitment, stability and activity of some chromatin regulators [162]. Particularly, OGT-mediated *O*-GlcNAcylation stabilizes TET1 [163] and OGT interacts preferentially with TET1 at gene promoters in close proximity of CpG-rich transcription start sites [164]. The multiprotein complex is recruited at the promoter region of nuclear factor erythroid 2-related factor 2 (Nrf2), a major transcription factor driving antioxidant enzymes expression; the upregulation of the transcription factor would be responsible for 5-FU resistance [161] (Figure 4E; Table 1). A genome-wide mRNA expression profiling of the National Cancer Institute NCI-60 human tumor cell lines screen, comprising seven different colon cancer cell lines, revealed that OGT expression is negatively correlated with sensitivity to fluorodeoxyuridine monophosphate (FdUMP), the active metabolite of 5-FU responsible for TS inhibition (Figure 3; Table 1). However the authors did not found any correlation with fluorodeoxyuridine (FUdR), 5-FU, topotecan or irinotecan sensitivity [165]. In parallel, Temmink et al. analyzed genes expression of H630 colon cancer cell line resistant to trifluorothymidine (TFT), another thymidine analogue which shares the same 5-FU metabolic pathway to inhibit TS [166]. These resistant cells underexpressed thymidine kinase (TK) (Figure 3), human equilibrate nucleoside transporter (hENT also known as SLC29A) involved in anabolism of 5-FU and also OGT. A link between OGT expression and activation of anabolic pathways leading to formation of active FdUMP seems to occur. Inhibiting *O*-GlcNAcylation is a promising approach to improve the sensitivity of cancer cells to anti-cancer drugs. In the context of breast cancer, it has been shown that *O*-GlcNAcylation-increasing treatments reduce estrogen receptor α (ERα) expression and protect MCF-7 cells from death induced by tamoxifen, a chemical largely used as a partial antagonist of the estrogen receptor in ER positive breast cancers [167].

#### *O*-GlcNAc regulation of resistance biomarkers

No mechanistic studies have been carried out to understand precisely the role of OGT in chemotherapy cell response. However, several studies revealed that clinical biomarkers of drug resistance [41, 42] can be either directly or indirectly regulated by *O*-GlcNAcylation. Specificity protein 1 (Sp1) and E2F transcription factor-1 (E2F-1), two key transcription factors that regulate a myriad of genes involved in nucleotide synthesis such as those coding dihydrofolate reductase (DHFR), TK and TS are controlled by *O*-GlcNAcylation (Figures 3 and 4E). E2F-1 is sequestered and inactivated by binding to hypo-phosphorylated and *O*-GlcNAcylated Rb during early G1 phase of cell cycle [145]. On the other hand, Sp1 was found to be *O*-GlcNAcylated in HT-29 colon cancer cells but the biological effect of the glycosylation was not reported [168] (Figure 4E). In breast cancer cells, *O*-GlcNAcylation of Sp1 regulates its stability and its transcriptional activity [169, 170]. In parallel, phosphorylation of TS at Ser124 by casein kinase 2 α subunit (CK2α) decreases its catalytic activity [171] but by interacting with CK2α Thr344 phosphorylation, *O*-GlcNAcylation at Ser347 decreases the stability of the kinase [172] (Figure 4E). Therefore, *O*-GlcNAcylation of CK2α should indirectly increase catalytic activity of TS. Recently, *O*-GlcNAcylation of thymidine phosphorylase (TP), a key enzyme of 5-FU anabolism which catalyzes the formation of FUdR from 5-FU and deoxyribose-1-phosphate (dRib-1-P) (Figure 3), was highlighted by mass spectrometry analysis of *O*-GlcNAcylated proteins enriched on succinyl-wheat germ agglutinin (sWGA) beads. TP was found to be *O*-GlcNAcylated only in breast tumors compared to adjacent healthy tissues but the role of this glycosylation has not been studied yet [173] (Figure 4E). Finally, DNA topoisomerase 1 (Top1), the target of the irinotecan active metabolite SN-38 (Figure 3), is also modified by *O*-GlcNAcylation *in vitro* and *in vivo*; this PTM increases its supercoiled DNA helix relaxation activity [174]. Since it was reported a positive correlation between Top1 activity and irinotecan sensitivity [175], *O*-GlcNAcylation may increase irinotecan sensibility through modification of Top1.

## CONCLUSIONS AND FUTURE PERSPECTIVES

While the percentages of obese and diabetic individuals are progressing rapidly in industrialized countries, epidemiological data show these patients display an increased risk of colorectal cancer (CRC) [176] and relapse after chemotherapy treatment [177–179]. These disorders may increase the availability of precursors involved in the biosynthesis of the nucleotide sugar donor uridine-5’-diphosphate-N-acetylglucosamine (UDP-GlcNAc) through the hexosamine biosynthetic pathway (HBP) and deregulate glycosylation processes. Oppositely, physical activity which has a blood glucose lowering effect appears to reduce risk of CRC recurrence following surgery combined with adjuvant chemotherapy [180]. However, glycosylation is a wide group of post-translational modifications and aberrant protein glycosylation is a common feature associated with cancer status and progression [27]. Thus, sialyl lewis a (sLea also referred to carbohydrate antigen CA 19-9) glycan and carcinoembryonic antigen (CEA) glycoprotein are serological tumor markers used actually to monitor CRC progression and disease recurrence [38].

The perspectives on emerging *N*-glycan-related anticancer therapies, along with new insights and challenges, are also currently being studied [181]. Recently, lectins have emerged as important biomedical tools that have been used in the development of anticancer agents. Several lectins have been shown to have the ability to discriminate between normal and tumor cells as a result of their different glycosylation patterns. Furthermore, the specific binding of lectins to cancer cells was shown to trigger mechanisms that promote the death of these abnormal cells. The review of de Oliveira Figueirôa and co-authors details the importance of lectins-carbohydrates interactions in cancer therapy and diagnosis [182]. They examine the use of nanoparticles exhibiting lectins (liposomes, solid lipid nanoparticles and other polymers) for anticancer drug delivery. They also discuss the development of drug delivery systems (alginate/chitosan microcapsules, alginate beads) carrying some antitumor lectins. In both cases (lectin-conjugated polymers or encapsulated lectins), these new pharmaceutical strategies improve intracellular delivery, bioavailability and cell targetability leading to enhanced therapeutic index and less side effects. In a very recent work, as sialoglycans overexpose the surface of cancer cells, sialic acid binding lectins have been explored for targeting cancer cells specifically. The potential of magnetic nanoparticles functionalized with wheat germ lectin (WGA) conjugates, so-called nanomagnetolectins, was thus successfully used as apoptotic targetable agents *in vitro* and *in vivo* for prostate cancer [183]. In addition to conventional drug therapy, lectin-based nanoparticles could specifically deliver to cancer cells gene or RNA interference (RNAi)-based therapies targeting key enzymes involved in therapy response [184]. In parallel, therapeutic carbohydrate-based vaccines approaches [185] are developed to target cancer-associated glycans in CRC, such as CEA [186–188] or Thomsen-nouvelle antigens on mucin 1 (Tn-MUC1) [189]. Same innovative strategies are under development to break the immunotolerance linked to their embryonic origin and/or low expression level in normal tissues. Consequently, immune effectors can be recruited to kill cancer cells overexpressing these aberrant glycans. As highlighted in this review, because of evidences linking altered glycosylation and drug resistance in CRC, one could imagine to combine immunotherapy with standard drug therapy treatments to improve their efficiency. Targeting oncofetal glycans such as Leb, Ley or Thomsen-Friedenreich antigen (T antigen) could also be a promising approach because of their high cancer-specificity.

However, it was widely described that the success of chemotherapies collides with the appearance of highly drug-resistant cancer stem cells (CSC) carrying numerous molecular changes conferring them a capability to relapse as chemoresistant tumors [56]. A set of CSC biomarkers has been discovered but fail to be used in clinic because of their expression in adult stem cells. Therefore, identification and characterization of oncofetal stem cell markers, not expressed in adult tissue, becomes one of the more promising therapeutic strategy challenges for the highly specific CSC targeting. As previously reviewed [56], several studies that explored differential glycoproteins and glycolipids patterns between CSC and other cancer cells strongly suggest that the expression of short-chain *O*-glycans is correlated with CSC phenotypes. Ferreira et al., therefore came up with a novel model that includes evaluation of glycomics and glycoproteomics in comprehensive pan-omics approaches envisaging more effective treatment strategy incorporating accurate patient classification and therapy design.

The literature findings summarized in this review provide evidence that abnormal glycosylation is also involved in diverse anticancer therapy resistance mechanisms including apoptosis failure, signaling activation disruption, altered drug absorption and metabolism, and cell stemness acquisition. However, our knowledge on this field remains limited. Understanding specific glycosylation alterations involved in resistance to CRC therapy can lead to the development of better therapeutic strategies with new predictive biomarkers and targets combined with an adapted diet.

## Abbreviations

5-FU: 5-fluorouracil
α2FucT: α1,2-fucosyltransferase
α3/4FucT: α1,3/4-fucosyltransferase
β3GnT: β1,3-N-acetylglucosaminyltransferase
ABC: ATP binding cassette
C2GnT: core 2 β1,6-N-acetylglucosaminyltransferase
C3GnT: core 3 β1,3-N-acetylglucaminyltransferase
C4GnT: core 4 N-acetylglucosaminyltransferase
CA: carbohydrate antigen
CEA: carcinoembryonic antigen
CMP-Neu5Ac: cytidine-5’-monophospho-N-acetylneuraminic acid
CRC: colorectal cancer
CSC: cancer stem cell
DISC: death inducting signaling complex
EGFR: epidermal growth factor receptor
EMT: epithelial-mesenchymal transition
ER: *endoplasmic reticulum*
Gal: galectin
GalNAc: N-acetylgalactosamine
GlcNAc: N-acetylglucosamine
GnT: N-acetylglucosaminyltransferase
HBP: hexosamine biosynthetic pathway
Le: Lewis
OGA: *O*-GlcNAcase
OGT: *O*-GlcNAc transferase
poly-LacNAc: poly-N-acetyllactosamine
ppGalNAcT: polypeptide N-acetylgalactosaminyltransferase
PTM: post-translational modification
S: sialyl
SN-38: 7-ethyl-10-hydroxycamptothecin
ST3GalT: α2,3-sialyltransferase
ST6GalNAcT: α-GalNAc α2,6-sialyltransferase
ST6GalT: β-galactoside α2,6-sialyltransferase
T antigen: Thomsen-Friedenreich antigen
Tn antigen: Thomsen-nouvelle antigen
TRAIL: TNF-related apoptosis-inducing ligand
TS: thymidylate synthetase
UDP-GalNAc: uridine-5′-diphosphate-N-acetylgalactosamine
UDP-GlcNAc: uridine-5′-diphosphate-N-acetylglucosamine
VEGF: vascular endothelial growth factor
